# Epidemiology of *Listeria monocytogenes* prevalence in foods, animals and human origin from Iran: a systematic review and meta-analysis

**DOI:** 10.1186/s12889-018-5966-8

**Published:** 2018-08-23

**Authors:** Reza Ranjbar, Mehrdad Halaji

**Affiliations:** 1 Molecular Biology Research Center, Systems Biology and Poisonings Institute, Baqiyatallah University of Medical Sciences, Tehran, Iran; 2 Department of Microbiology, School of Medicine, Isfahan University of Medical Sciences, Isfahan, Iran

**Keywords:** *Listeria monocytogenes*, Food pathogen, Listeriosis, Meta-analysis, Iran

## Abstract

**Background:**

*Listeria monocytogenes* as the main causative agent of human listeriosis is an intracellular bacterium that has the capability to infect a wide range of cell types. Human listeriosis is a sporadic foodborne disease, which is epidemiologically linked with consumption of contaminated food products. Listeriosis may range from mild and self-limiting diseases in healthy people to severe systemic infections in susceptible populations. This study aimed to investigate the prevalence of *L. monocytogenes* in food resources and human samples from Iran.

**Methods:**

A systematic search was performed by using electronic databases from papers that were published by Iranian authors Since January of 2000 to the end of April 2017. Then, 47 publications which met our inclusion criteria were selected for data extraction and analysis by Comprehensive Meta-Analysis Software.

**Results:**

The pooled prevalence of *L. monocytogenes* in human origin was 10% (95% CI: 7–12%) ranging from 0 to 28%. The prevalence of *L. monocytogenes* in animals was estimated at 7% (95% CI: 4–10%) ranging from 1 to 18%. Moreover, the pooled prevalence of *L. monocytogenes* in Iranian food samples was estimated at 4% (95% CI: 3–5%) ranging from 0 to 50%. From those 12 studies which reported the distribution of *L. monocytogenes* serotypes, it was concluded that 4b, 1/2a, and 1/2b were the most prevalent serotypes.

**Conclusions:**

The prevalence of *L. monocytogenes* and prevalent serotypes in Iran are comparable with other parts of the world. Although the overall prevalence of human cross-contamination origin was low, awareness about the source of contamination is very important because of the higher incidence of infections in susceptible groups.

**Electronic supplementary material:**

The online version of this article (10.1186/s12889-018-5966-8) contains supplementary material, which is available to authorized users.

## Background

*Listeria* is ubiquitous Gram-positive bacteria, which are rod-shaped, facultative anaerobic, and non-spore forming, with a low C + G content [1]. The genus *Listeria* is composed of several species, of which *Listeria monocytogenes* is an opportunistic pathogen of humans and animals [1]. Due to ubiquitous nature of *Listeria* spp., and their unique ability to survive across a broad range of environmental stress including pH, temperature, and salt, they are considered as important foodborne pathogens [2].

*L. monocytogenes* as the main causative agent of human listeriosis is an intracellular bacterium that has the capability to infect a wide range of cell types and cross the intestinal, blood-brain and placental barriers [3]. Human listeriosis is a sporadic foodborne disease, which is epidemiologically linked with consumption of contaminated food products [4]. In human, listeriosis may range from a mild and self-limiting flu-like sickness or febrile gastroenteritis in healthy people to severe systemic infections including meningitis, septicemia, and abortion in susceptible people [3]. High-risk individuals are the pregnant women, neonates, elderly, immunocompromised individuals and adults with malignancy [5]. Listeriosis can be a serious disease with an approximate 20% mortality; that case–fatality rate may increase in groups at highest risk [4]. Regarding the wide distribution of *L. monocytogenes* in food resource and high fatality rate of listeriosis, *L. monocytogenes* has been considered as a major public health concern [1].

Variation in Iranian food tastes results in consumption of different kinds of foods, which may be considered as a risk factor of listeriosis outbreaks. Despite some local information on the prevalence of *Listeria* spp. in various food resources in Iran, there is no comprehensive data available on its prevalence to estimate the burden of *L. monocytogenes*. Therefore, this study aimed to investigate the prevalence of *L. monocytogenes* in food resources and human samples from Iran by using a systematic review and meta-analysis based method. This finding can provide good epidemiological background contributing to the international data of *L. monocytogenes* distribution.

## Methods

### Search strategies

A systematic literature search was conducted in the Web of Science, PubMed, Scopus and Google Scholar electronic databases from papers that were published by Iranian authors since January of 2000 to the end of April 2017. The following terms, “Listeriosis” or “*Listeria*” or “*L. monocytogenes*”, in combination with “Food”, “Animal”, “Human”, and “Iran” were searched as scientific keywords in the present survey both separately and simultaneously in March and April 2017.

### Selection criteria and quality assessment

Two reviewers independently screened the databases with the related keywords and reviewed the titles, abstracts, and full texts to determine the articles which met the inclusion criteria; any discrepancies were resolved by consensus. The articles published in English or Persian language with English abstract which indexed in Pubmed or Scopus and had met the inclusion criteria were considered in our survey: standard methods (Culture methods, the results based on antibodies (ELISA) and molecular techniques) were used for *Listeria* detection, present data on the prevalence of *L. monocytogenes*, and samples were collected from foods or clinical samples. The criteria for identifying Iranian authors were the author or location of the work and also affiliations of authors. Additionally, research that has been conducted by non-Iranian authors on the Iranian population or samples were also assessed. Studies that did not use standardized methods, the sample size was less than 10 isolates, duplicate reports, and articles, samples obtained from environment sources or the origin of samples was unclear in them, articles that were written in Persian with Persian abstract and studies which did not detect *L. monocytogenes* were excluded. The quality of eligible studies was judged independently by two authors in accordance with the Joanna Briggs Institute. Eventually, the studies that obtained more than 60% were included in this study [6].

### Data extraction

The following details were extracted for each of the included studies: the first author’s name, the time of performing the study, publication date, the study setting, sample size, source of isolation, the frequency of *Listeria* spp., and *L. monocytogenes* serotypes.

### Statistical analysis

To estimate the overall prevalence meta-analyses, “metaprop program” in STATA version 14.0 (STATA, College Station, TX, USA) statistical software was used [7]. Meta-analysis was performed by using the random-effects model to estimate the pooled prevalence and corresponding 95% confidence interval (CI). Statistical heterogeneity groups were estimated using the Cochran Chi-square test and the Cochrane-I2. The funnel plot, Begg’s rank correlation test, and Egger’s weighted regression tests were used to evaluate possible publication bias (*P* < 0.05 was considered as an indication of a statistically significant publication bias). Possible sources of heterogeneity were evaluated by sensitivity analysis, meta-regression and subgroup analysis based on the location of the study and diagnostic methods [8, 9]. Sensitivity analysis was applied to determine that the exclusion of any study has a significant effect on the estimated pooled prevalence while ignoring each individual one. The present study designed according to the Preferred Reporting Items for Systematic Reviews and Meta-Analyses (PRISMA) guidelines (Additional file 1).

## Results

**Fig. 1:**
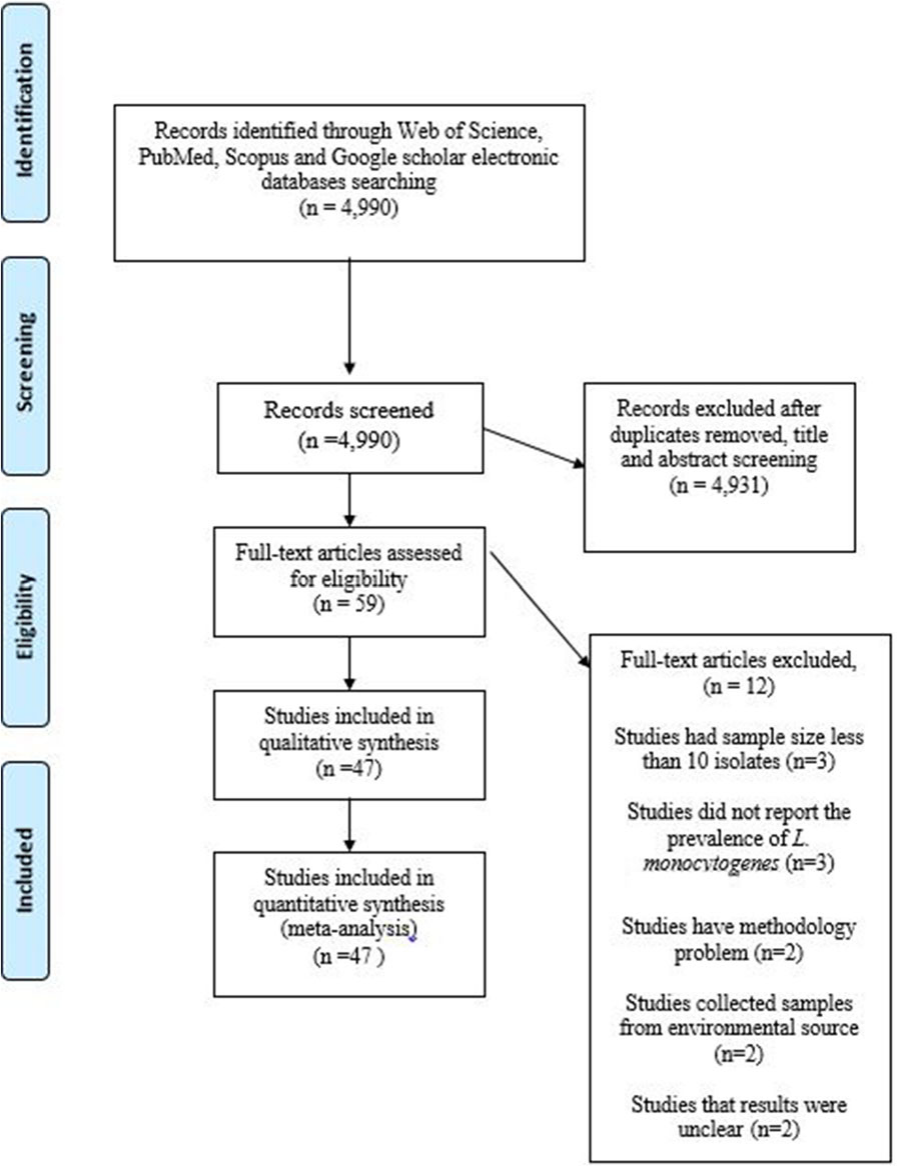
Flow chart of study selection for inclusion in the systematic review

The database search yielded 4990 citations. Among them, 4931 were removed by index, title and abstract screening and 59 were accessed in full text. Of 59 reviewed studies, three studies had a sample size less than 10 isolates, three studies did not report the prevalence of *L. monocytogenes*, two studies had a methodological problem, two studies collected samples from environment sources, and results of two studies were unclear. Finally, 47 studies matched with eligibility criteria and were subjected to meta-analysis, [2–4, 10–53]. However, out of 47 included studies, three studies reported prevalence in animals and/ humans and/food, simultaneously. The searching procedure for selection of eligible studies is demonstrated in Fig. 1.

The full results of the included articles, sample size, the prevalence of *L. monocytogenes* and predominant serotypes are presented in Table 1.

**Table 1:** Characteristics of studies included in the meta-analysis

Author	Publication year	Years of study	City or Province/Region	Diagnostic method	Sample source	Types food/animal species/Human	Study design	Sample size	*L. monocytogenes*	Predominant serotypes	*L. innocua*	Total	Ref
Firouzi et al.	2000	UN	Shiraz/South	Culture	Human	Slaughter houses	Cross sectional	130	0	–	0	3	6
Akhondzadeh Basti et al.	2004	UN	Tehran and Guilan/North	Culture	Food	Fresh fish, salted and smoked fish	Cross sectional	120	4	4b	–	–	7
Moshtaghi et al.	2007	2005	Shahrekord/Central	Culture	Food	Raw milk	Cross sectional	500	8	4b	3	11	8
Rahimi et al.	2008	2006–2007	Isfahan/Central	Culture	Animal	Cattle	Cross sectional	200	6	–	–	–	9
Jalali et al.	2008	2003–2005	Isfahan/ Central	PCR	Food	Meat, diary, vegetables products ready to eat food	Cross sectional	461	7	–	13	27	4
Jamshidi et al.	2009	2002–2003	Bandar Abbas/South	Serology	Human	‘Spontaneous abortion, control group	Case-control study	450	124	–	–	–	10
Jami et al.	2010	2008	Mashhad/Northeast	PCR	Food	Raw milk	Cross sectional	100	4	–	–	–	11
Rahimi et al.	2010	2007–2009	Isfahan/Central	PCR	Food	Milk,dairy products	Cross sectional	594	18	–	32	55	12
Mahmoodi et al.	2010	UN	Noorabad/South	Culture	Food	Raw milk, White cheese, yoghurt	Cross sectional	360	6	–	–	–	13
Lotfollahi et al.	2011	2009–2010	Tehran/North	Culture	Human	Spontaneous abortions	Cross sectional	100	9	–	–	–	14
Rahimi et al.	2011	UN	Tehran/North	Culture	Human	Pregnant mothers	Cross sectional	512	5	–	–	–	15
Rahimi et al.	2011	2009–2010	Isfahan and Shahrekord/Central	PCR	Food	Sea food	Cross sectional-	264	5	–	15	20	16
Ghasemian Safaei et al.	2011	2008	Shahrekord/Central	Culture	Food	Eggs	Cross sectional	100	0	–	–	–	17
Goudarzi et al.	2012	2011	Karaj/North	PCR	Human	Women with septic abortion	Cross sectional	87	12	–	–	–	18
Fallah et al.	2012	2010–2011	Shahrekord/Central	PCR	Food	Poultry product	Cross sectional	402	52	4b, 1/2a, 1/2b, 1/2c	62	134	2
Zarei et al.	2012	UN	Ahvaz/Southwest	PCR	Food	Raw/fresh,frozen, and ready-to-eat (RTE) seafood	Cross sectional	245	2	–	–	–	19
Hosseinzadeh et al.	2012	2009	Shiraz/South	PCR	Animal	Poultry flocks	Cross sectional	100	7	–	–	–	20
Safarpoor Dehkordi et al.	2012	2011	Various parts of Iran	Real-Time PCR	Food	Milk	Cross sectional-	596	69	–	–	–	21
					Animal	Vaginal swab/Urine samples		1575	158				
Rahimi et al.	2012	2009–2010	Chahar Mahal & Bakhtiyari/Central and South	Culture and PCR	Food	Dairy products	Cross sectional	290	5	–	14	21	22
Rahimi et al.	2012	2010–2011	Various parts of Iran	PCR	Food	Raw meats	Cross sectional	1107	27	–	98	141	23
Seifi et al.	2012	2009–2010	North and west	PCR	Animal	Broiler fl ocks	Cross sectional	490	44	–	–	–	24
Fallah et al.	2013	2011–2012	Shahrekord/ Central	PCR	Food	Raw and RTE seafood product	Cross sectional	462	35	1/2a, 4b, 1/2c, 1/2b, 4c	–	–	25
Jamali et al.	2013a	2008–2010	Tehran/North	PCR	Food	Raw milk	Cross sectional	446	18	1/2a, 3a; 1/2c, 3c; 4b, 4d, 4e	48	83	26
Jamali et al.	2013b	2008–2010	Tehran/North	PCR	Food	Milk	Cross sectional	207	17	(4b, 4d or 4e), (1/2a or 3a), (1/2b, 3b or 7), (1/2c or 3c)	3	21	27
Sohrabi et al.	2013	UN	Isfahan/Central	Culture	Food	Poultry meat	Cross sectional	52	1	–	11	12	28
Vahedi et al.	2013	2011	Sari/North	Culture	Food	Milk	Cross sectional	200	0	–	–	–	29
Momtaz et al.	2013	2010–2011	Isfahan and Shahrekord/Central	PCR	Food	Fresh fish/shrimp samples	Cross sectional	300	18	4b, 1/2b, 1/2a	2	24	30
Salehian et al.	2013	2012	Sari/North	Culture	Food	Traditional ice cream	Cross sectional	50	1	–		–	31
Zarei et al.	2013	UN	Ahvaz/ Southwest	PCR	Food	Beef, buffalo and lamb meats	Cross sectional	210	7	–	–	–	32
Akya et al.	2013	UN	Kermanshah/West	Culture	Food	Dairy, meat, products,RTE	Cross sectional	530	3	–	56	66	33
Shakib et al.	2013	UN	Lorestan/West	PCR	Human	Pregnant women	Cross sectional	100	0	–	–	–	34
Rahimi et al.	2014	2010–2011	Fars and Khuzestan/South and Southwest	PCR	Food	Bulk milk, camel, Water, buffalo, ovine, caprine,	Cross sectional	260	7	–	13	27	3
Eslami et al.	2014	2012–2013	Tehran/North	PCR	Human	Women with abortion	Cross sectional	96	16	–	–	–	35
Alidoosti et al.	2014	2012	Isfahan/Central	Culture	Animal	domestic dogs		92	1	1/2b	–	–	36
Jamali et al.	2014	2008–2010	Tehran/North	Culture	Animal	Duck, goose intestinal contents	Cross sectional	471	19	–	5	58	37
Moosavy et al.	2014	UN	Tabriz/Northwest	Culture	Food	Raw milk	Cross sectional	18	9	–	–	–	38
Haghi et al.	2015	2014	Zanjan/West	PCR	Food	Bovine milk, ovine milk	Cross sectional	60	0	–	–	–	39
Haghroosta et al.	2015	UN	Ahvaz/ Southwest	Serologic	Human	Pregnant women with spontaneous abortion and healthy pregnant women	Cross sectional	180	43	–	–	–	40
Jamali et al.	2015	2012–2014	Mazandaran/North	PCR	Food	Raw fish	Cross sectional	488	104	1/2a, 4b, 1/2b	34	37	41
Soltan Dallal et al.	2015	2013	Tehran/North	Culture	Food	Vegetables, salads	Cross sectional	200	1	–	–	48	42
Mashak et al.	2015	2011–2012	Tehran/North	Culture	Food	Fresh, frozen meats	Cross sectional	410	115	1/2a, 4b, 4c, 3b	–	–	43
Mansouri-Najand et al.	2015	2011	Kerman/Central	PCR	Food	Raw milk	Cross sectional	100	5	–	–	–	44
Pourkaveh et al.	2016	2015	Tehran/North	PCR	Human	Women with spontaneous abortion	Cross sectional	317	54	–	–	–	45
Abdollahzadeh et al.	2016	2014–2015	Karaj and Tehran/North	PCR	Food	Fish, shrimp, RTE seafood	Cross sectional	201	5	1/2b, 3b, 7, 1/2a, 3a	–	18	46
Pournajaf et al.	2016	2012–2015	Tehran/North	PCR	Human	Patients with spontaneous abortions	Cross sectional	170	14	–	–	–	47
					Food	Dairy products/meat,		317	20				
					Animal	Domestic animals		130	12				
Zeinali et al.	2017	2013	Mashhad/North east	PCR	Animal	Fresh chicken carcasses	Cross sectional	200	36	–	23	80	48
Lotfollahi et al.	2017	2013–2015	Tabriz/North west	PCR	Food	Sausage, milk, cheese, chicken, meat	Cross sectional	267	8	(1/2c or 3c), (4b, 4d or 4e), (1/2a or 3a)	–	–	49
					Human	Pregnant woman with a abortion		125	11				
					Animal	Goat and sheep carcasses		50	3				

**Fig. 2:**
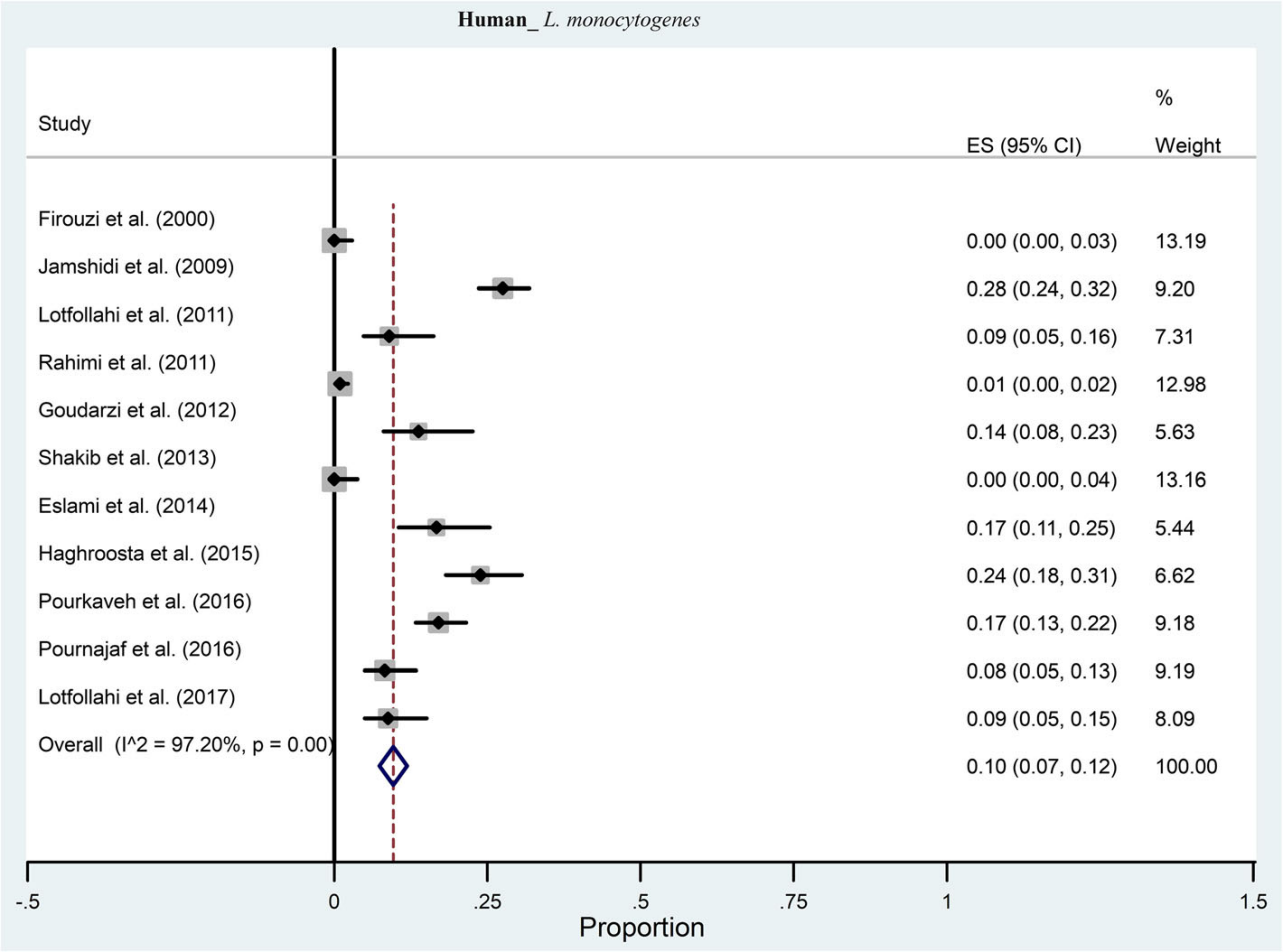
Forest plot of the meta-analysis of *L. monocytogenes* prevalence in human

Eleven studies investigated the prevalence of *L. monocytogenes* in humans. From those studies, the pooled prevalence of *L. monocytogenes* was 10% (95% CI: 7–12%) ranging from 0 to 28% (Fig. 2). There was a significant heterogeneity among the 11 studies (χ2 = 331.98; *p* < 0.001; I^2^ = 97.2%). The funnel plot for publication bias showed evidence of asymmetry. Additionally, Begg’s and Egger’s tests were performed to quantitatively evaluate the publication biases. According to the results of Begg’s test (z = 1.48, *p* = 0.02) and Egger’s test (*t* = 5.21, *p* < 0.001) a significant publication bias was observed.

**Fig. 3:**
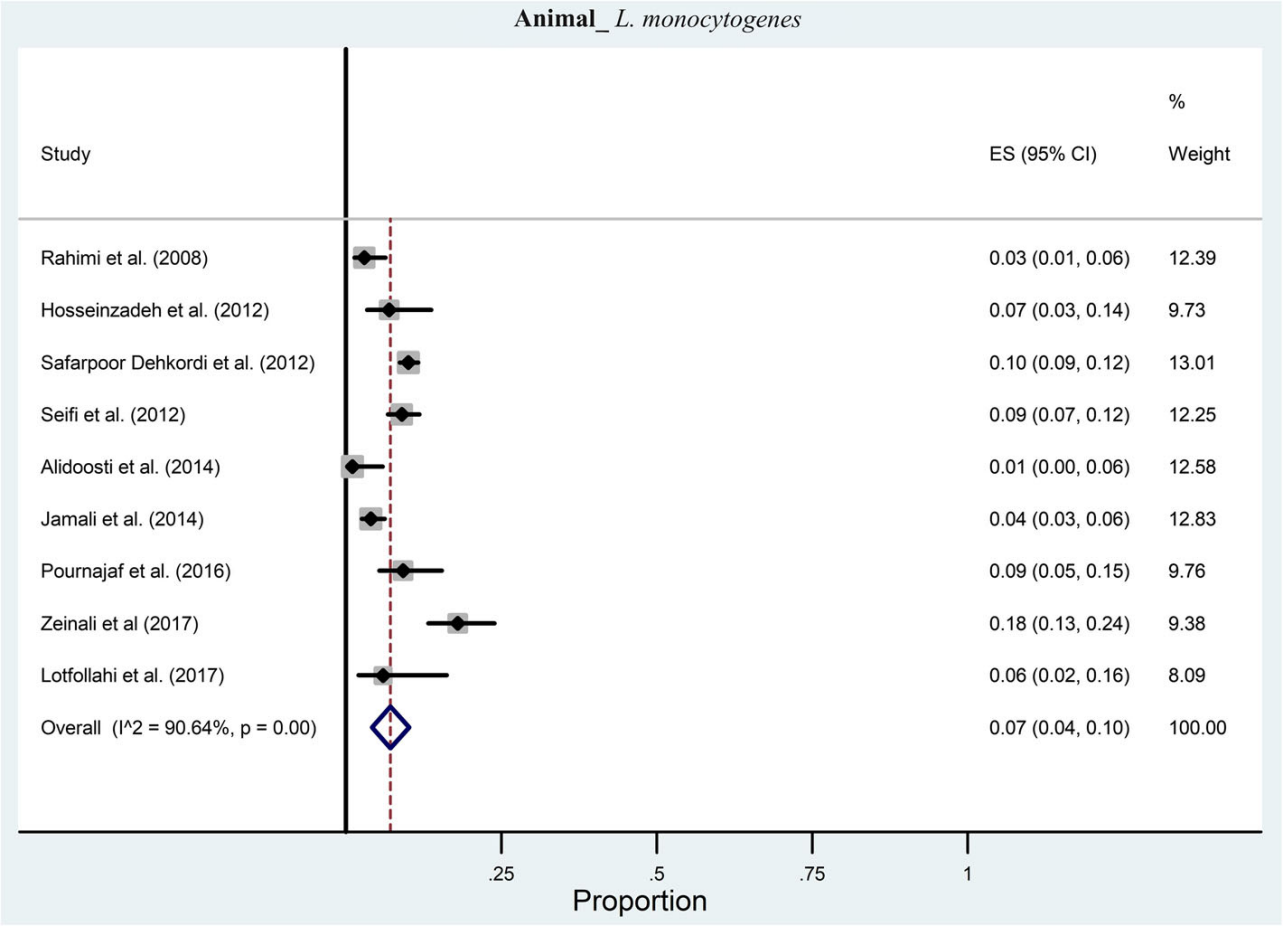
Forest plot of the meta-analysis of *L. monocytogenes* prevalence in animals

According to the included publications, in nine studies the prevalence of *L. monocytogenes* was investigated in animals. The pooled prevalence of *L. monocytogenes* was estimated at 7% (95% CI: 4–10%) ranging from 1 to 18% (Fig. 3). There was a significant heterogeneity among the nine studies (χ2 = 85.46; *p* < 0.001; I^2^ = 90.64%). The symmetric funnel plot showed no evidence of publication bias and confirmed by the results of Begg’s test (z = 0.21, *p* = 0.835) and Egger’s test (*t* = 1.62, *p* = 0.116).

**Fig. 4:**
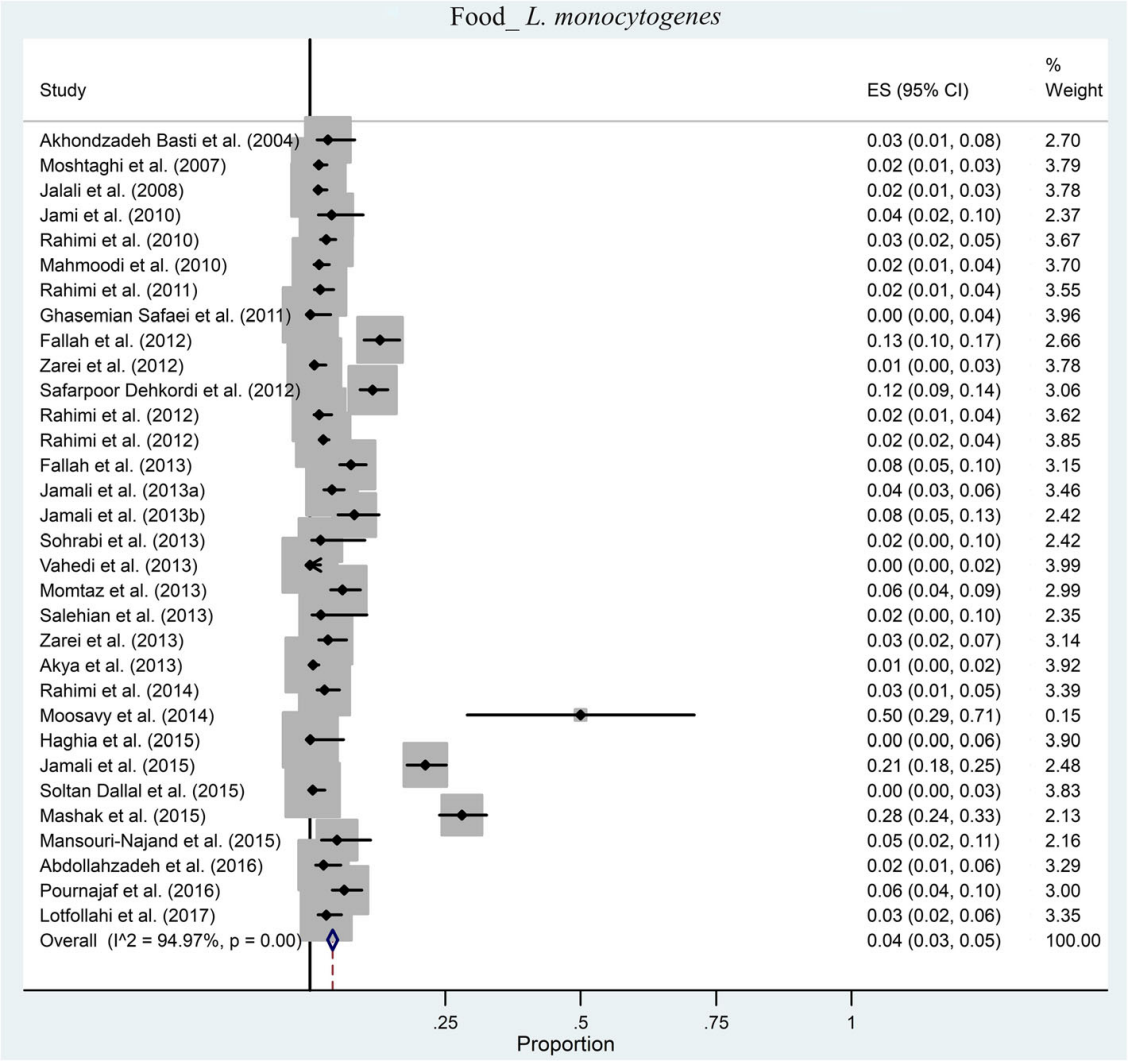
Forest plot of the meta-analysis of *L. monocytogenes* prevalence in foods

**Fig. 5:**
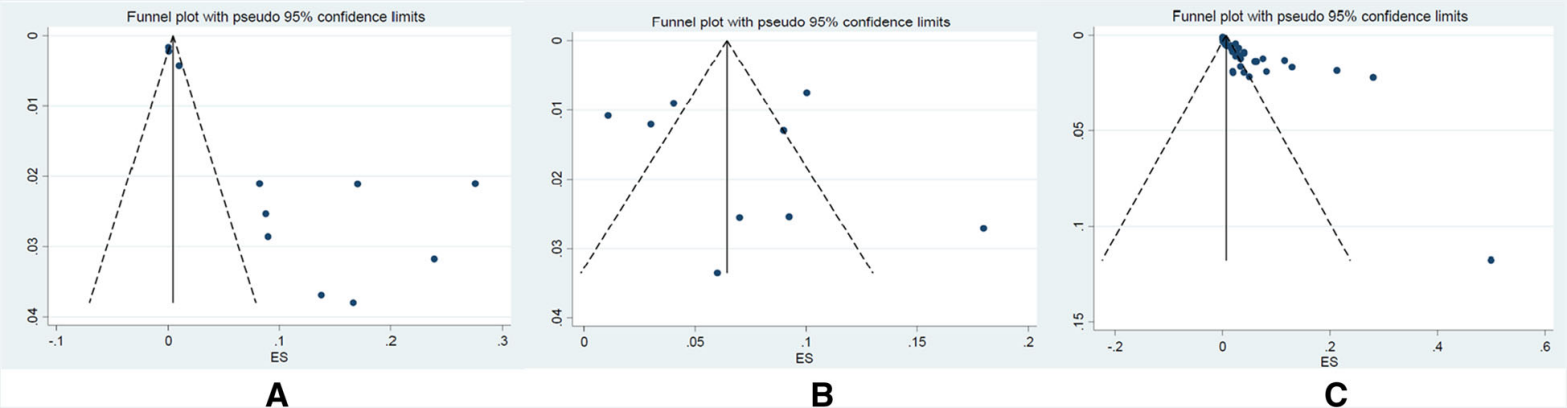
Funnel plot of publication bias for the included studies related to **a** Human, **b** Animal, and **c** Food

We found 32 articles which investigated the prevalence of *L. monocytogenes* in foods samples. The pooled prevalence of *L. monocytogenes* in Iranian food samples was estimated at 4% (95% CI: 3–5%) ranging from 0 to 50% (Fig. 4). Based on Q statistic and the I^2^ index heterogeneity was significant (χ2 = 573.757; *p* < 0.001; I^2^ = 94.97%). There was evidence of strong publication bias from the funnel plot of the included articles (Fig. 5); it was confirmed by Begg’s rank correlation analysis (z = 3.73, *p* < 0.001). However, Egger’s regression analysis showed a significant publication bias (*t* = 1.62, *p* = 0.116).

Of the totally included articles, only in 12 studies the distribution of *L. monocytogenes* serotypes was reported. From those studies, it was concluded that 4b, 1/2a, and 1/2b were the most prevalent serotype. Furthermore, the pooled prevalence of *L. inocua* was 5.6% ranging from 4.1 to 7.7%.

The results of subgroup analysis based on geographic location in human samples showed that pooled prevalence of *L. monocytogenes* was 14% (95% CI: 1–36%; *n* = 3 studies), 10% (95% CI: 4–18%; *n* = 7 studies), and 1% (95% CI: 0–5%; *n* = 1 studies) in South, North (West and East) and West of Iran, respectively (Additional file 2: Figure S1). The results of subgroup analysis based on diagnostic methods in human samples showed that pooled prevalence of *L. monocytogenes* was 1% (95% CI: 0–3%; *n* = 3 studies), 26% (95% CI: 23–30%; *n* = 2 studies), and 11% (95% CI: 4–17%; *n* = 6 studies) based on culture, serology and PCR methods, respectively (Additional file 2: Figure S1).

The results of subgroup analysis based on geographic location in food samples showed that pooled prevalence of *L. monocytogenes* was 7% (95% CI: 4–10%; *n* = 13 studies), 4% (95% CI: 2–5%; *n* = 11 studies), and 2% (95% CI: 1–3%; *n* = 4 studies), 3% (95% CI: 3–4%; *n* = 2 studies) in North (West and East), Central, South and all parts of Iran, respectively (Additional file 2: Figure S1).

The results of subgroup analysis based on the diagnostic methods in food samples showed that pooled prevalence of *L. monocytogenes* was 3% (95% CI: 1–4%; *n* = 11 studies), 5% (95% CI: 3–6%; *n* = 19 studies), and 12% (95% CI: 9–14%; *n* = 1 studies), 2% (95% CI: 1–4%; *n* = 1 studies) based on culture, PCR, Real-Time PCR and culture and serology methods, respectively (Additional file 2: Figure S1).

The results of subgroup analysis based on geographic location in Animal samples showed that pooled prevalence of *L. monocytogenes* was 9% (95% CI: 5–13%; *n* = 5 studies), 2% (95% CI: 0–4%; *n* = 2 studies), and 7% (95% CI: 3–14%; *n* = 1 studies) in North (West and East), Central and South of Iran, respectively (Additional file 2: Figure S1). The results of subgroup analysis based on the diagnostic methods in Animal samples showed that pooled prevalence of *L. monocytogenes* was 10% (95% CI: 6–13%; *n* = 5 studies) and 3% (95% CI: 1–5%; *n* = 3 studies) based on PCR and methods, respectively (Additional file 2: Figure S1).

### Sensitivity analysis and meta-regression

The sample size of included studies could not be accounted as the causes of heterogeneity due to the result of carried meta-regression analysis in which no possible associated effect was observed between a sample size of included studies and pooled prevalence.

Besides, sensitivity analysis’s results concluded that none of the incorporated studies has the ability to change the overall prevalence substantially (Additional file 3: Figure S2).

## Discussion

Direct transmission of *L. monocytogenes* from the infected animals or contaminated raw products is the main route of human cross-contamination [54]. The unique ability of this microorganism to survive food preservation or hostile environments and the presence of numerous bacterial surface components and extracellular virulence factors make *L. monocytogenes* as a serious threat to food safety [1, 55]. To the best of our knowledge, this study is the first comprehensive systematic review of the prevalence of *L. monocytogenes* in foods, animal and human origin from Iran, simultaneously. Based on the meta-analysis results, the overall estimate of *L. monocytogenes* prevalence among human origin with 10% was slightly higher than animal and food resources, i.e. 7% and 4%, respectively. However, some reasons may explain the higher prevalence of *L. monocytogenes* in Iranian population compared to environmental sources. First, most of the human origin studies were performed on susceptible groups including pregnant women or hospitalized patients, so the burden of *L. monocytogenes* infections would be expected to be lower in the general community. Second, in two studies with the highest isolation rate, authors used the serological method for detection of *L. monocytogenes* among the participants [14, 44] because antigenic cross-reactivity serological methods have lower discriminatory power in epidemiological studies compared to molecular methods [56].

Due to the multifactorial nature of *L. monocytogenes* prevalence, its international comparison is challenging. It seems that some factors have more profound effects on the prevalence of *L. monocytogenes*. Regarding the role of sample type, with some variation incidence of *L. monocytogenes* contamination in dairy products tends to be lower than other resources such as vegetables or meat products (mostly less than 10%) [57–64]. Based on previous reports, the infection rate of domestic and wild animals is frequently higher than foods origin and has a much more variation [65–71].

Gain a global estimate of *L. monocytogenes* infections in human is even more challenging since most of the studies looking in the distinct range of society or samples [72–78]. Besides the variation according to the origin of isolation, various incidence rates of *L. monocytogenes* may arise from differences in the sample size, seasonal variability, and geographical distribution.

*Listeria innocua* is a ubiquitous non-pathogenic membrane of genus *Listeria*. This bacterium does not seem to carry the virulence-associated genes described in pathogenic species [79]. However, recently it has been shown that *L. innocua* can invade bovine trophoblasts, but it is unable to multiply in the intracellular environment [80]. In our findings the isolation rate of *L. innocua* among Iranian food resources was remarkable. To date, there is no report of human complication by this bacterium from Iran; however, two cases of *L. innocua* human infections were reported in European countries [79, 81]. These observations make us keep in mind that we should not rule out the potential risk of *Listeria* contamination rather than *L. monocytogenes*.

Analysis of the included studies revealed serotypes 4b, 1/2a, and 1/2b as the most prevalent serotypes. From annual trends of serotypes changes, it seems that 4b serotype is losing its dominant position and replaced by 1/2a and 1/2b. However, serotypes can be variable during different time periods, seasons or geographical distributions, and different sample type. Wang et al. showed 648 food samples collected within years 2013–2014 in Shanghai, China the majority of the isolates (more than 80%) belonged to serotypes 1/2a, and 1/2b [58]. Kevenk et al. from Turkey reported the presence of four different serotypes (1/2a, 1/2b, 1/2c, and 4b) in isolates obtained from milk and dairy products [61]. Haley et al. showed the predominance of 3 serogroups (1/2a, 1/2b, and 4b) in the isolates collected during 2004 and 2010 within a U.S. dairy herd [82]. In a study on several regions of Brazil from 1975 to 2013, with the same serotype distribution, Almeida and colleagues introduced 4b, 1/2b, and 1/2c, as the main serotypes in human and food sources [83]. Serotypes l/2a, l/2b, and 4b were the most prevalent serotypes in sows and fattening pigs in France in 2008 [70]. Hasegawa et al. showed the predominance of 1/2b, 1/2a, and 4b serotypes among black beef cattle in Japan [68]. Surveillance of invasive listeriosis within the years 2006–2010 in Italy, revealed serotypes 1/2a, 4b, and 1/2b as the frequent types [84]. When rank correlation methods show bias, the bias is likely evidence of small studies effect [85]. Meanwhile, meta-regression analysis showed that weight of studies could not be considered as a confounding factor. Also, sensitivity analysis on included studies indicated that exclusion of any study has no significant effect on the estimated pooled prevalence.

The limitations of our systematic review include the following: Firstly, due to the extent of *L. monocytogenes* has not yet been examined in many regions of Iran, we cannot fully represent the frequency of *L. monocytogenes* in the country. Secondly, the studies could not fully indicate the prevalence of *L. inocua* in Iran, because the prevalence of *L. inocua* has not yet been surveyed in many studies conducted in Iran. Third, heterogeneity was detected among the included studies therefore, the results should be interpreted with caution.

## Conclusions

The results of the present study provide good epidemiological information about the contamination status and distribution of *L. monocytogenes* among Iranian resources. The prevalence of *L. monocytogenes* and prevalent serotypes in Iran is comparable with other parts of the world. Although the overall prevalence of human cross-contamination source was low, awareness about the source of contamination is very important because of a higher incidence of infections in susceptible groups.

## Additional files

Additional file 1: Study design according to the Preferred Reporting Items for Systematic Reviews and Meta-Analyses (PRISMA) guidelines. (DOC 57 kb)

Additional file 2: **Figure S1.** Forest plot of pooled estimated prevalence of *L. monocytogenes* in subgroup analysis based on geographic location and diagnostic methods in Human (1), Food (2) and Animal samples (3). (ZIP 7589 kb)

Additional file 3: **Figure S2.** Sensitivity plot of studies included in the systematic review and meta-analysis related to (a) Human, (b) Animal, and (c) Food. (ZIP 248 kb)

## Abbreviations

CI: Confidence interval
PRISMA: Preferred Reporting Items for Systematic Reviews and Meta-Analyses
