# Supplementary figures and images for: Epidemiology of Listeria monocytogenes prevalence in foods, animals and human origin from Iran: a systematic review and meta-analysis
Source: BMC Public Health. 2018 Aug 23;18:1057. doi: 10.1186/s12889-018-5966-8 (PMC6108140; doi:10.1186/s12889-018-5966-8)

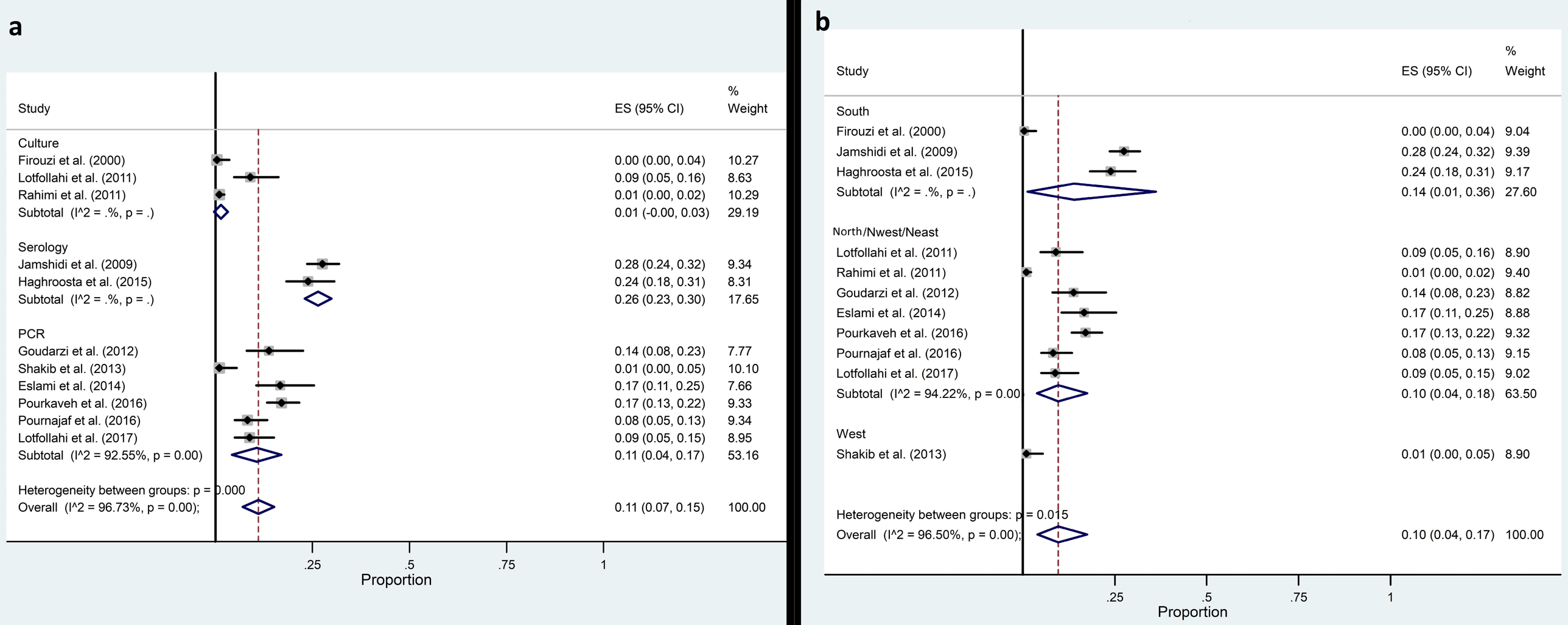

Supplement: Supplementary file 2 — Figure S1. Forest plot of pooled estimated prevalence of L. monocytogenes in subgroup analysis based on geographic location and diagnostic methods in Human (1), Food (2) and Animal samples (3). (ZIP 7589 kb) [file 12889_2018_5966_MOESM2_ESM.zip › Figure 1 (1) S1R4.jpg]

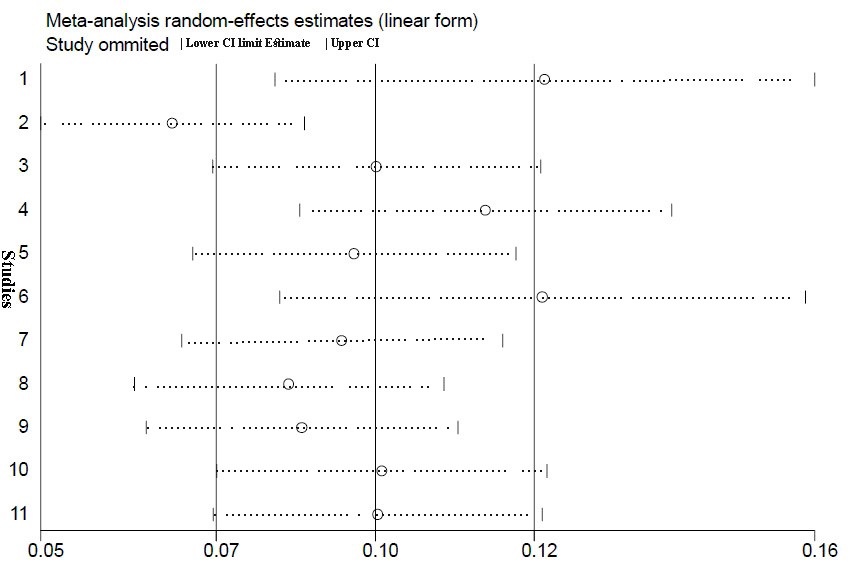

Supplement: Supplementary file 3 — Figure S2. Sensitivity plot of studies included in the systematic review and meta-analysis related to (a) Human, (b) Animal, and (c) Food. (ZIP 248 kb) [file 12889_2018_5966_MOESM3_ESM.zip › Figure 2 (a)S1R4.jpg]

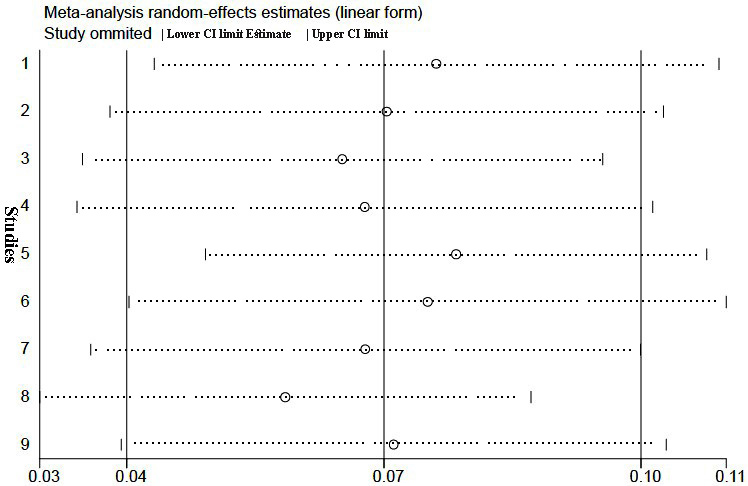

Supplement: Supplementary file 3 — Figure S2. Sensitivity plot of studies included in the systematic review and meta-analysis related to (a) Human, (b) Animal, and (c) Food. (ZIP 248 kb) [file 12889_2018_5966_MOESM3_ESM.zip › Figure 2 (b) S1.jpgR4.png]

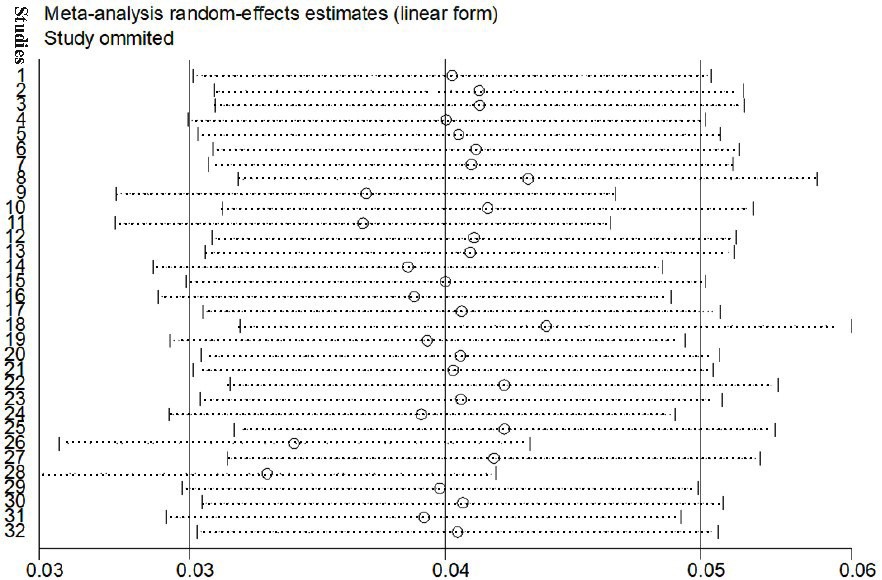

Supplement: Supplementary file 3 — Figure S2. Sensitivity plot of studies included in the systematic review and meta-analysis related to (a) Human, (b) Animal, and (c) Food. (ZIP 248 kb) [file 12889_2018_5966_MOESM3_ESM.zip › Figure 2 (c) S1R4.jpg]
